# Supplementary material for: Mistaking imagination for reality: Congruent mental imagery leads to more liberal perceptual detection
Source: Cognition. 2021 Jul;212:104719. doi: 10.1016/j.cognition.2021.104719 (PMC8164160; doi:10.1016/j.cognition.2021.104719)
Supplement: Supplementary file 1 — Supplementary material [file mmc1.docx]

**Abstract.** Visual experiences can be triggered externally, by signals coming from the outside world during perception; or internally, by signals from memory during mental imagery. Imagery and perception activate similar neural codes in sensory areas, suggesting that they might sometimes be confused. In the current study, we investigated whether imagery influences perception by instructing participants to imagine gratings while externally detecting these same gratings at threshold. In a series of three experiments, we showed that imagery led to a more liberal criterion for reporting stimulus presence, and that this effect was both independent of expectation and stimulus-specific. Furthermore, participants with more vivid imagery were generally more likely to report the presence of external stimuli, independent of condition. The results can be explained as either a low-level sensory or a high-level decision-making effect. We discuss that the most likely explanation is that during imagery, internally generated sensory signals are sometimes confused for perception and suggest how the underlying mechanisms can be further characterized in future research. Our findings show that imagery and perception interact and emphasize that internally and externally generated signals are combined in complex ways to determine conscious perception.

**Keywords:** mental imagery, perception, reality monitoring, signal detection theory

**1. Introduction**

In daily life, we are bombarded with visual input from the outside world. Different shapes, colours and textures are processed by our visual system to create the technicolour perception we experience every day. At the same time, while we are caught up in thinking about past or future events, our brain internally generates a rapid stream of mental images (Delamillieure et al., 2010). Various lines of research have shown that externally triggered perception and internally triggered mental imagery activate similar neural codes in sensory as well as high-level brain areas (for reviews, see Dijkstra, Bosch, & van Gerven, 2019; Pearson, 2019), even in the presence of external input (Rademaker et al., 2019; but see also Bettencourt & Xu 2015). This leads to the hypothesis that engaging in imagery might influence how the brain processes external inputs during perception.

An interaction between mental imagery and perception has been investigated within different contexts. One line of research has shown that imagining one of two interpretations of an ambiguous stimulus prior to its presentation increases the probability of subsequently perceiving that same stimulus (Chiou, Rich, Rogers, & Pearson, 2018; Keogh & Pearson, 2011, 2014, 2017; Pearson, Clifford, & Tong, 2008; Sherwood & Pearson, 2010). However, a recent study found large between-subject variation in this effect with imagery leading to strong adaptation effects in some participants (Dijkstra, Hinne, Bosch, & van Gerven, 2019). These results can be explained by the idea that imagery functions as a top-down prior, biasing perception towards or away from the imagined percept by pre-activating stimulus-specific neural populations, similar to the effects of expectation (Denison, Piazza, & Silver, 2011; Dijkstra, Hinne, Bosch, & van Gerven, 2019; Hohwy, Roepstorff, & Friston, 2008).

Another line of research has investigated the influence of imagery on the detection of unambiguous but near-threshold external stimuli. Some studies found that simultaneous imagery increased the likelihood that an external stimulus was detected (Moseley, Smailes, Ellison, & Fernyhough, 2016; Saad & Silvanto, 2013; Segal & Fusella, 1970) whereas others found an imagery-induced decrease in the detection of external stimuli (Okada & Matsuoka, 1992; Perky, 1910; Segal & Nathan, 1964). These findings have both been interpreted as cases of source confusion: mistaking an imagined signal for an external stimulus, or vice versa. Misattributing an imagined signal to the external world would lead to an imagery-induced increase in external presence reports (“Yes, there was a stimulus”) (Moseley et al., 2016) whereas misattributing an external signal to one’s imagination would lead to an imagery-induced decrease in presence reports (“No, I imagined that stimulus”) (Okada & Matsuoka, 1992; Perky, 1910). Alternatively, these effects may result from imagery affecting how well external signals are processed, rather than any misattribution of signal source. Specifically, increases in presence responses could arise from imagery lowering the detection threshold for imagined stimuli, akin to effects of expectation (Chang, Kanai, & Seth, 2015; Pinto, van Gaal, de Lange, Lamme, & Seth, 2015; Stein & Peelen, 2015) or even by creating a response bias towards saying ‘present’. Decreases in presence responses on the other hand could be the result of imagery causing general distraction effects via a decrease in processing capacity (Segal & Fusella, 1970).

In the current study, we combined near-threshold psychophysics with large, robust sample sizes afforded by web-based data collection to investigate how imagery affects external perceptual detection. In a series of experiments, we investigated the influence of imagery on signal detection theoretic measures of detection and whether this effect was influenced by expectation, was stimulus-specific and whether individual differences in imagery vividness played a role. Participants detected gratings at threshold while simultaneously imagining either the same gratings, orthogonal gratings, or nothing at all. If imagery merely causes non-specific cognitive effects, such as distraction or a general bias to respond ‘present’, we would expect it to affect detection rates independent of whether the imagined and detected stimuli were congruent or not. In contrast, if imagery was sometimes confused for perception, or vice versa, we would expect imagery to bias presence reports in a stimulus-specific way. Furthermore, the direction of this confusion might depend on the likelihood of stimulus presentation: participants might be more likely to confuse imagery for perception when they expect stimulus presence and more likely to confuse perception for imagery when they expect stimulus absence. Finally, if this effect is indeed specific to imagery rather than a general top-down effect we would expect a correlation with imagery vividness.

**2. Experiment 1**

**2.1 Materials and Methods**

**2.1.1. Participants.** 150 participants (mean age = 25.8, *SD* = 8.3) were recruited using Prolific ([www.prolific.co](http://www.prolific.co)) and completed the study online. Data were collected on a private institutional server managed by the JATOS tool (Lange, Kühn, & Filevich, 2015). Informed consent was obtained from each participant included in the study. The study took approximately 40 minutes to complete and participants were paid £5 for their contribution, equivalent to an hourly rate of £7.50. All procedures were approved by the University College London ethics committee. Data from 13 participants were not obtained due to technical issues. Furthermore, we excluded experimental blocks if (1) detection accuracy was below 50% or above 95% or (2) the imagery check was incorrect (see below). We excluded participants if they had fewer than 2 usable blocks (48 trials) in each condition. This led to the exclusion of 41 participants. In total, 96 participants were included in the final analysis.

**2.1.2. Experimental procedures and design.** The experimental paradigm is depicted in Figure 1. A participant’s task was to detect gratings in dynamic noise while either imagining these same gratings or not. Prior to the main experiment, participants filled out two questionnaires: the vividness of visual imagery questionnaire (VVIQ2; Marks, 1973; 1995) and the Revised Launay–Slade Hallucination Scale (LSHS-R; Launay & Slade, 1981; McCarthy-Jones & Fernyhough, 2011; Morrison, Wells, & Nothard, 2000). After this, participants first practiced detecting full-contrast stimuli in noise until they responded correctly on at least 75% of the trials, making sure they understood the task. Then the threshold visibility of the gratings (leading to 70% accuracy) was determined via a staircase procedure, separately for the two orientations. Finally, participants practiced imagining the gratings while looking at dynamic noise for twenty trials, indicating the vividness of their imagery after each trial using a scale from 1 (not vivid at all) to 5 (perfectly clear and as vivid as real seeing), similar to the scale used in the VVIQ.

In order to avoid visual priming, no trial-wise cues were delivered. Instead, the different conditions were implemented in a block-wise fashion. At the onset of each experimental block, the participant was instructed which grating orientation would be shown and whether or not they should also imagine this grating during the block (Fig. 1) as follows: “During this block you will see right/left tilted gratings. Please also/do not imagine this grating during each trial.” To continue to the main block, participants had to press the space bar. The order of the blocks was randomized within each participant. Each trial started with a 200ms fixation cross followed by 2 seconds of either pure dynamic white noise or dynamic white noise within which a gradually appearing stimulus was embedded. The task of the participants was to indicate whether or not a grating was present on each trial. The base rate of presence versus absence was 50/50 but participants were told that a grating would be present in 75% of the trials. This was done to increase the number of false alarms (experiment 2 shows that the imagery effect was not dependent on this instruction). After each block, participants were asked whether or not they imagined the stimulus, to ensure that they had correctly followed the instructions. Blocks were removed prior to analyses if the answer to this imagery check was incorrect. There were twelve blocks in total, consisting of 24 trials each.


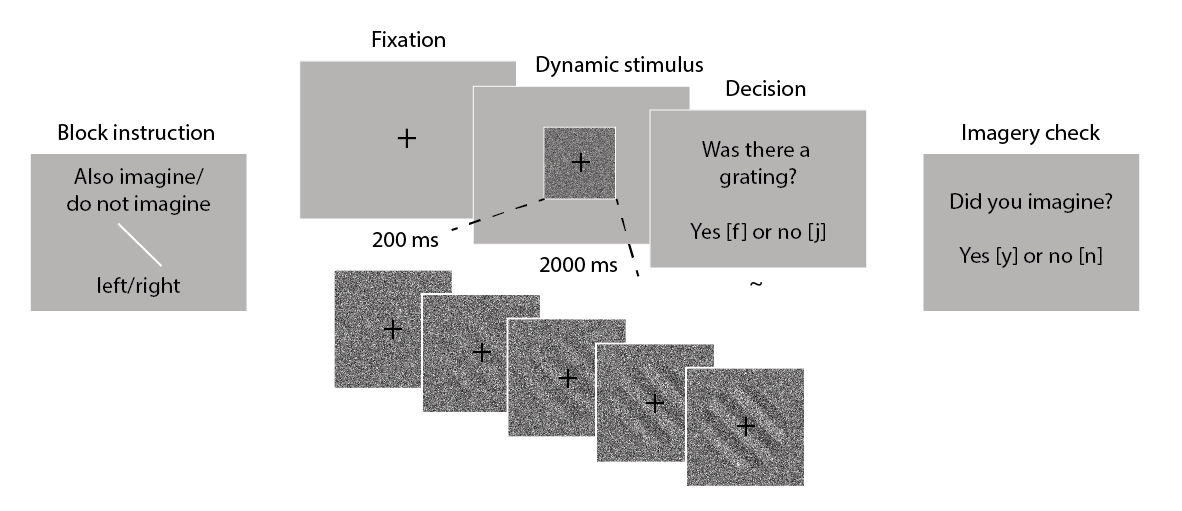


**Figure 1. Experimental paradigm.** At the start of each block, participants were instructed whether they would be detecting left or right tilted gratings and whether they would have to imagine those gratings during this block. Trials consisted of 200ms fixation followed by 2s of either a grating ramping up in noise (50%) or pure noise (50%). Participants had to indicate whether a grating was present or not. After each block participants were asked if they did or did not imagine the grating during this block to check whether they followed instructions.

The stimuli were generated in MATLAB (version R2018b) and consisted of sinusoidal gratings tilted at an orientation of 45^o^ or 135^o^, masked with an annulus and embedded in white noise (Fig. 1). The visibility of the stimuli was manipulated by changing the probability that a given pixel was replaced by a random value. For each orientation separately, stimulus images of 50 visibility levels were generated. Furthermore, for the absence trials, 20 images of pure white noise were generated. The main experiment was programmed in JavaScript using jsPsych (de Leeuw, 2015). The threshold visibility level of each orientation was determined per participant in a staircase procedure prior to the main experiment. The staircases contained 120 trials each and accuracy was calculated after every 10 trials. Visibility was increased if accuracy was below 65 and decreased if accuracy was above 75.

During stimulus-present trials, twenty stimulus images ranging from zero visibility to 70% detection threshold were presented over the course of 2 seconds, giving the impression that the stimulus was gradually ramping up. This ramping up was done to mimic the gradual nature of mental image generation (Perky, 1910). During stimulus-absent trials, twenty noise images were presented in random order.

**2.1.3. Data analysis.** We used signal detection theory to analyse the data (Ingleby, 1967). Detection sensitivity (*d’*) and criterion (*c*) were calculated separately for the imagery and no-imagery trials as follows:

$d^{'}=z\left( H \right)-z\left( FA \right)$

$c=-0.5 \times\left[ z\left( H \right)+z\left( FA \right) \right]$

where $z$ indicates the inverse of the cumulative normal distribution, $H$ is the hit rate (the proportion of present trials for which the participant reported presence), and $FA$ is the false alarm rate (the proportion of absent trials for which the participant reported presence). Detection sensitivity $d^{'}$ is a measure of detection performance, with greater values indicating better performance. Criterion $c$ is a measure of participant’s bias towards responding ‘yes’ (present) or ‘no’ (absent), irrespective of whether a stimulus is present or not. Greater values of $c$ indicate a more conservative criterion, indicating a greater tendency towards reporting absence. Hit rates of 1 or false alarm rates of 0 lead to biased estimations of $d^{'}$ and$c$. To correct for this, in those cases of extreme values we added a count of 0.5 to the relevant cell (Hautus, 1995).

**2.2. Results**

The results of experiment 1 are shown in Figure 2. Criterion $c$ was significantly lower in the imagery condition (*M* = -0.061, *SD* = 0.524) compared to the no-imagery condition (*M* = 0.264, *SD* = 0.511; *t*(95) = 7.693, *p* = 1.33e^-11^), indicating a higher tendency to report stimulus presence during imagery. Theoretically, criterion and detection sensitivity are independent, so a change in criterion does not necessarily lead to a change in$d^{'}$. However, detection sensitivity $d^{'}$ was also significantly lower in the imagery condition (*M* = 1.581, *SD* = 0.701) compared to the no-imagery condition (*M* = 1.815, *SD* = 0.655; *t*(95) = 3.933, *p* = 0.0002) indicating that performance was also worse during imagery. This effect on $d^{'}$ can be explained by the fact that the imagery-induced increase in false alarm rate (*M* = 0.112, *SD* = 0.151) was greater than the increase in hit rate (*M* = 0.060, *SD* = 0.132; *t*(95) = 3.169, *p* = 0.002). Together, these results indicate that imagining a stimulus leads to an increase in the tendency to report the stimulus as present, especially when the stimulus was actually absent.


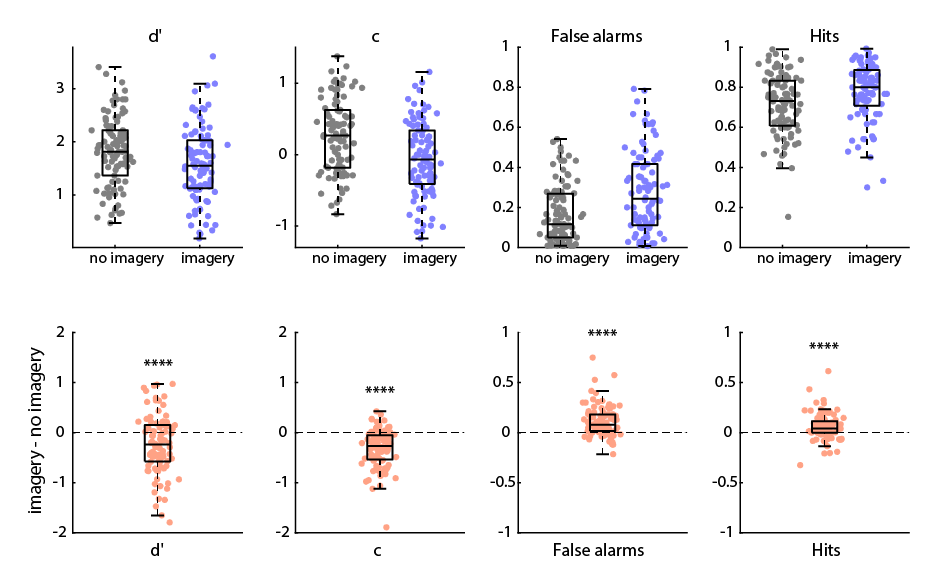


**Figure 2. Main effect of imagery on SDT measures in Experiment 1.** Top row: values per condition. Grey is without imagery and blue is with imagery. Bottom row: without imagery – with imagery. Positive difference indicates greater values during imagery and negative difference indicates smaller values during imagery compared to no imagery. Dots represent individual participants. Boxplot lines represent the range of data excluding outliers. **** p < 0.0001.

We also investigated individual differences in SDT measures. We calculated Spearman correlations to be more sensitive to potential non-linear effects in our data and to be less sensitive to outliers. There were no significant correlations between any of the questionnaire scores and any of the SDT measures (all p-values > 0.07). However, we did observe strong significant correlations between several SDT measures and mean imagery vividness ratings during the imagery practice trials. There were 12 SDT measures ($d^{'}$,$c$,$FA$ and $H$ per condition and difference between conditions). We corrected for multiple comparisons using a Bonferroni correction such that only correlations with a *p*-value lower than 0.05/12 = 0.0042 were considered significant.

There was no significant correlation between vividness and $d^{'}$ during the no-imagery condition (*r* = -0.238, *p* = 0.019), imagery condition (*r* = -0.277, *p* = 0.006) or the difference between them (*r* = 0.088, *p* = 0.39). There was a significant negative correlation between imagery vividness and criterion $c$ during both the no-imagery (*r* = -0.356, *p* = 0.0004) and the imagery condition (*r* = -0.458, *p* = 0.000003) but not the difference between them (*r* = 0.109, *p* = 0.29). There was a significant positive correlation between imagery vividness and $FA$ during both the no-imagery (*r* = 0.392, *p* = 0.00008) and the imagery condition (*r* = 0.449, *p* = 0.000005) but not the difference between them (*r* = -0.239, *p* = 0.019). Finally, there was also a significant positive correlation between imagery vividness and $H$ during the imagery condition (*r* = 0.314, *p =* 0.002) but not the no-imagery condition (*r* = 0.215, *p* = 0.035) or the difference between them (*r =* -0.07, *p* = 0.5)*.* Together, these results indicate that higher imagery vividness was associated with a more liberal criterion and increase in false alarm rate during both imagery and no-imagery conditions, despite there being no relationship between vividness and the magnitude of the effect of imagery on detection.

In summary, this first experiment showed that imagining a stimulus increased the tendency to report seeing that stimulus, and that imagery vividness also positively correlated with the tendency to report external presence. One explanation for these results is in terms of source confusion: during imagery blocks participants sometimes confused their imagery for external stimulation, leading to an increase in presence reports. However, we did not observe the reverse: external stimulation sometimes being confused for imagery, leading to an imagery-induced increase in misses. One reason for this might be that participants were more likely to attribute imagery to perception than vice versa because there was an expectation of external stimulus presence (‘if I see something, it is probably real’). In contrast, if external stimulus presence is less likely, perception might be more likely to be attributed to imagery (‘if I see something, it is probably imagined’). In the next study we investigate whether the imagery effect is influenced by such expectations.

**3. Experiment 2**

**3.1. Materials and Methods**

**3.1.1. Participants.** 150 participants were recruited and data were collected in the same way as in experiment 1 (see Section 2.1.1). Data from 12 participants were not obtained due to technical issues. Experimental blocks were excluded based on the same criteria as in experiment 1. Due to the addition of an experimental factor (expectation) and the resulting lower number of blocks per condition, we now excluded participants if they had less than 1 usable block (24 trials) in each condition. This led to the exclusion of 40 participants. Together, 98 participants were included in the final analysis (mean age = 29.4, *SD* = 10.54).

**3.1.2. Experimental procedures and design.** The main experimental design was similar to experiment 1 (Fig. 1). In contrast to experiment 1, we did not obtain VVIQ and LHS questionnaire responses here because the previous experiment showed that these did not correlate with the experimental measures. Furthermore, in this experiment we additionally manipulated expectations of presence and absence by changing the base rate of presentation, again in a block-wise fashion. In the expect-absence condition the base rate was 20% (higher probability of absence) whereas in the expect-presence condition the base rate was 80% (higher probability of presence). In contrast to experiment 1, we showed the correct base rate in the instructions to participants such that they were more likely to form accurate expectations as follows: “During this block you will see right/left tilted gratings. Please also/do not imagine this grating during each trial. There will be a grating present on 20/80% of the trials.” We hypothesized that expecting presence would also decrease the criterion in addition to any effect of imagery. There were 3 blocks per condition (expectation x imagery) with 24 trials each.

**3.1.3. Data analysis.** SDT measures were calculated as in experiment 1 separately for each of the four conditions; expectation (presence/absence) x imagery (no-imagery/imagery).

**3.2. Results**

The results of experiment 2 are shown in Figure 3. A repeated-measures ANOVA with imagery and expectation as within-subject factors and the SDT measures as dependent measures was conducted. We again observed a significant main effect of imagery on criterion $c$ with a lower criterion in the imagery (*M* = 0.320, *SD* = 0.346) compared to the no-imagery condition (*M* = 0.529, *SD* = 0.308; *F*(92,1) = 29.578, *p* < .0001, *eta^2^* = .243), replicating the findings of experiment 1. There was also a main effect of expectation on $c$ with a lower criterion in the expect-presence (*M* = 0.246, *SD* = 0.347) compared to the expect-absence condition (*M* = 0.602, *SD* = 0.370; *F*(92,1) = 53.497, *p* < .0001, *eta^2^* = .368). However, there was no significant interaction between imagery and expectation (*F*(92,1) = 0.891, *p*= 0.348), indicating that the effect of imagery was not influenced by expectation. Furthermore, in contrast to experiment 1, in experiment 2 there was no significant main effect of imagery on $d^{'}$ (*F*(92,1) = 3.200, *p*= 0.077). There was also no significant main effect of expectation on $d^{'}$ (*F*(92,1) = 0.130, *p*= 0.719) or an interaction between these two factors (*F*(92,1) = 0.611, *p*= 0.437).


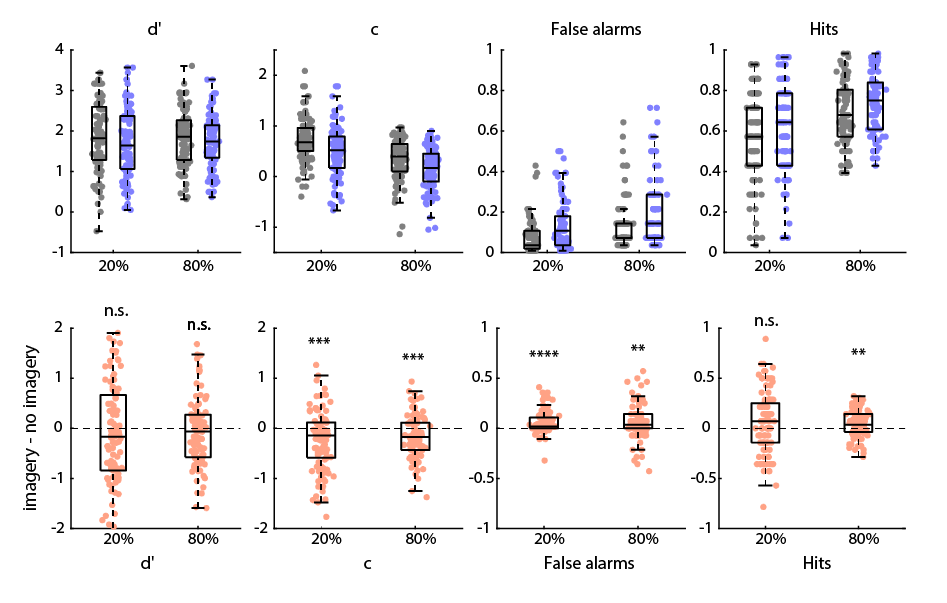


**Figure 3. Main effect of imagery on SDT measures in Experiment 2.** Top row: values per condition (imagery x expectation). Grey is without imagery and blue is with imagery; a base-rate of 20% represents an expectation of absence and a base-rate of 80% represents an expectation of presence. Bottom row: difference between imagery and no-imagery blocks for the two expectation conditions. Positive difference indicates greater values during imagery and negative difference indicates smaller values during imagery compared to no imagery. ** p < 0.01, *** p < .001, *** p < 0.0001, **** p < 0.00001

We again investigated individual differences in SDT measures by correlating them with the mean imagery vividness ratings obtained during the imagery practice trials. Because we did not observe any significant correlations between vividness and the difference between imagery and no-imagery in experiment 1 and because the effects were similar in both imagery conditions, here we investigated SDT measures calculated over all trials together. We performed 4 tests, giving a Bonferroni corrected p-value cut-off of 0.05/4 = 0.0125. Similar to experiment 1, there was no significant correlation between imagery vividness and $d^{'}$ (*r* = -0.189, *p* = 0.062) but there was a significant negative correlation between vividness and $c$ (*r* = -0.327, *p* = 0.001). Furthermore, there was a significant positive correlation between vividness and$FA$ (*r* = 0.323, *p* = 0.001), but not between vividness and $H$ (*r* = 0.124, *p* = 0.225). Together, these findings suggest that the more vivid participants’ imagery was, the more likely they were to respond present during the detection task, especially when there was no actual stimulus present.

To investigate whether participants’ awareness of imagery influencing their responses was related to the magnitude of the imagery effect, we asked participants at the end of the experiment “Do you feel like imagining the gratings influenced your responses on the task?”. Participants were categorized into three categories based on their responses: ‘Yes’ (responses containing ‘yes’ or ‘I do’; *N* = 51), ‘Maybe’ (responses containing ‘maybe’, ‘a little’, ’slightly’, ’possibly’, ’perhaps’, ’some’; *N* = 21) and ‘No’ (responses containing ‘no’; *N* = 26). A repeated-measures ANOVA with expectation and imagery as within-subject variables and awareness of influence as a between-subject variable revealed a significant interaction between imagery and awareness on $c$ (*F*(92,2) = 3.560, *p* = 0.033, eta^2^ = 0.073): the effect of imagery on $c$ was largest for ‘yes’ responders (*M* = 0.291, *SD* = 0.381), smaller for ‘sometimes’ responders (*M* = 0.205, S*D* = 0.360) and smallest for ‘no’ responders (*M* = 0.049, *SD* = 0.320) . This shows that as a group, participants had metacognitive insight into the effect of imagery on their visual detection responses: those participants who showed the strongest effects on the decision criterion also tended to notice it and respond ‘yes’.

In summary, the second experiment demonstrated that an imagery-induced increase in presence responses was independent of expectation. Furthermore, we again observed a positive correlation between imagery vividness and general detection criterion, indicating that more vivid imagery is associated with a higher likelihood of responding presence during external stimulus detection. These results could again be explained by source confusion – if people mistake their imagery for real stimuli, they will be more likely to say stimuli are present when they are also engaging in imagery. However, in both experiments, the imagery condition differed from the no-imagery condition in more aspects than only the presence of internally activated mental images. Compared to the no-imagery condition, imagery is associated with an increase in cognitive control, internal attention and executive function (Kosslyn, 2001; Pearson, 2019). These dual-task demands could have (partly) caused the changes in external detection behavior reported here. To control for this possibility, we next investigated whether the imagery effects were stimulus-specific and only present when the imagined and detected stimuli were congruent. In this case, the only difference between the congruent and incongruent condition is the content of the mental image. If the effects were due to the general dual-task nature of the imagery condition, we should also observe them for both congruent and incongruent imagery.

**4. Experiment 3**

**4.1 Materials and Methods**

**4.1.1. Participants.** Based on the size of the effects in the previous two experiments, we performed a power calculation to determine the number of participants for this experiment. Assuming a medium effect of 0.5, 34 participants would be required to reach a power of 80% (Dhand & Khatkar, 2014). Taking into account drop-out, 40 participants were recruited and data were collected in the same way as in experiment 1 (see Section 2.1.1). Data from 3 participants were not obtained due to technical issues. Experimental blocks were excluded based on the same criteria as in the previous experiments. In this experiment, there were 4 blocks per condition (no-imagery, congruent imagery & incongruent imagery and we excluded participants if they had less than 2 useable blocks in each condition (48 trials). This led to the exclusion of 1 participant. In total, 36 participants were included in the final analysis (mean age = 30.94, *SD* = 13.59).

**4.1.2. Experimental procedures and design.** The main experimental design was similar to experiment 1 (Fig. 1). In this experiment we additionally manipulated stimulus congruency by including a condition in which participants were instructed, at the start of the block, to imagine a grating with orthogonal orientation to the one they were detecting during that block as follows: “During this block you will see right/left tilted gratings. Please do not imagine/also imagine left tilted gratings/also imagine right tilted gratings during each trial.” To check whether participants accurately followed instructions, after each block we asked whether participants imagined nothing, a left tilted grating or a right tilted grating. We hypothesized that only congruent imagery would increase presence responses (i.e., decrease$c$).

**4.2 Results**

The results for experiment 3 are shown in Figure 4. Criterion $c$ was significantly reduced during congruent imagery (*M* = 0.286 , *SD* = 0.660) compared to no-imagery (*M* = 0.511, *SD* = 0.554; *t*(35) = -2.966, *p* = 0.0054), but not during incongruent imagery (*M* = 0.550 , *SD* = 0.771) compared to no-imagery (*t*(35) = 0.474, *p* = 0.639). Accordingly, the imagery-effect on $c$ was larger for congruent (*M* = -0.225, *SD* = 0.456) than incongruent imagery (*M* = 0.039, *SD* = 0.495; *t*(35) = -3.018, *p* = 0.0047). In contrast to experiment 1 but in line with experiment 2, there was no significant difference in $d^{'}$ during congruent imagery (*M* = 1.977 , *SD* = 0.666) compared to no-imagery (*M* = 2.008 , *SD* = 0.708; *t*(35) = -0.311, *p* = 0.758). However, there was a significant decrease in $d^{'}$ during incongruent imagery (*M* = 1.485, *SD* = 0.807) compared to no-imagery (*t*(35) = -5.458, *p* = 0.000004), indicating that incongruent imagery hampered performance. Accordingly, the imagery-effect on $d^{'}$ was larger for incongruent (*M* = -0.523, *SD* = 0.575) compared to congruent imagery (*M* = -0.031, *SD* = 0.595; *t*(35) = -3.994, *p* = 0.0003). This double dissociation between the effect of congruent and incongruent imagery on $c$ and $d'$ is caused by the different effects on false alarm and hit rates. While there was no significant difference in the imagery-induced increase in false alarms between congruent (*M* = 0.053, *SD* = 0.138) and incongruent imagery (*M* = 0.054, *SD* = 0.126; *t*(35) = -0.034, *p* = 0.973), hit rate was increased during congruent imagery (*M* = 0.063, *SD* = 0.145) but decreased during incongruent imagery (*M* = -0.086, *SD* = 0.179; *t*(35) = 4.517, *p* = 0.00007), leading to a decrease in sensitivity for incongruent imagery and a decrease in criterion for congruent imagery.


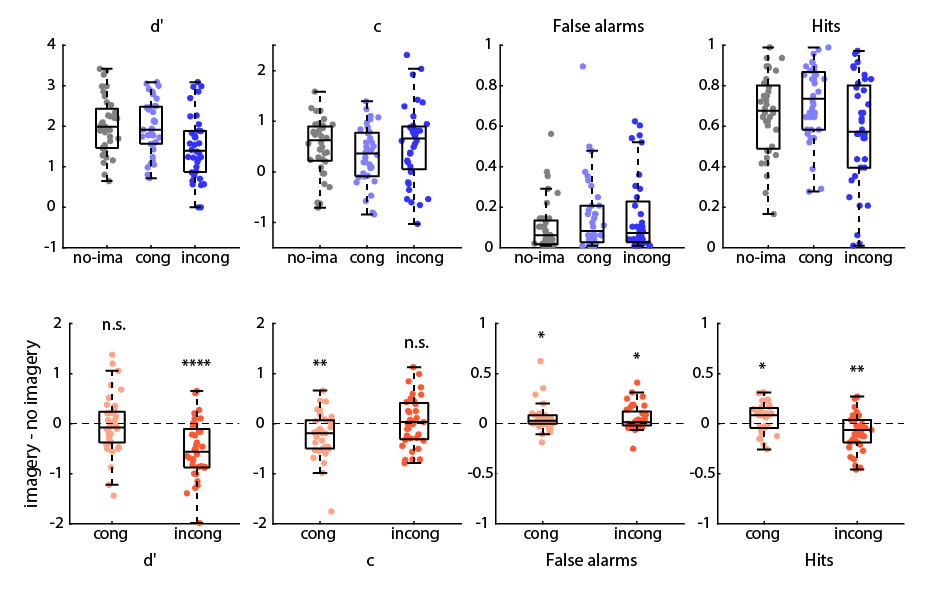


**Figure 4. Main effect of imagery on SDT measures in Experiment 3.** Top row: values per condition. Grey is without imagery; blue is with congruent imagery and red is with incongruent imagery. Bottom row: difference between imagery and no-imagery blocks for congruent and incongruent imagery. Positive difference indicates greater values during imagery and negative difference indicates smaller values during imagery compared to no imagery. * p < 0.05 ** p < 0.01, *** p < .001, *** p < 0.0001, **** p < 0.00001

Together, these results indicate that the imagery-induced decrease in criterion is specific to the content of the mental image. In contrast, a decrease in d’ might be partly due to the dual-task nature of the imagery compared to no-imagery conditions. Therefore, the double dissociation between $c$ and $d^{'}$ can be explained by source confusion only occurring during congruent imagery, when the internal and external stimuli are similar. Under this explanation, during congruent imagery participants confuse their mental image to reflect perception, leading to a heightened tendency to report stimuli are present. This is unlikely to occur during incongruent imagery, when participants know that any external stimuli would be orthogonally oriented to the one they are imagining. In contrast, the decrease in $d^{'}$ during incongruent imagery may have been caused by imagery disrupting the processing of congruent input, leading to both an increase in false alarms together with a decrease in hits. This explanation would mean that an increase in $FA$ observed in the two imagery conditions (Fig. 4) is caused by different mechanisms (source confusion for congruent imagery and distraction for incongruent imagery) and should therefore not correlate. To test this, we calculated the Spearman correlation between the imagery effects on $FA$ for congruent and incongruent imagery. There was a significant positive correlation (*r* = 0.358, *p* = 0.032), suggesting that there is a relationship between the mechanisms underlying these effects and potentially challenging either the source confusion and distraction account. However, it could also be that participants sometimes thought they perceived the imagined stimuli during incongruent imagery, such that the $FA$ effect here actually also represented source confusion. Indeed, in experiment 2 a few participants reported that, despite the instructions, they believed the other orientation was also sometimes presented. Alternatively, both source confusion and distraction could be driven by the strength of mental imagery as a third common factor, with stronger imagery leading to both more source confusion and more distraction.

We again investigated the relationship between imagery vividness and external signal detection. In line with experiment 1 and 2, there was a significant negative correlation between imagery vividness and$c$ (*r* = -0.525, *p* = 0.001), indicating that more vivid imagery was correlated with a higher tendency to report presence. However, in contrast to the previous findings, we also observed a significant negative correlation between vividness and $d^{'}$ (*r* = -0.511, *p* = 0.001), suggesting that more vivid imagery was also associated with worse detection performance (possibly due to the effect of imagery on the size of the incongruent distraction effect). Furthermore, there was a strong significant positive correlation between vividness and$FA$ (*r* = 0.673, *p* = 0.00007), but not between vividness and $H$ (*r* = 0.278, *p* = 0.101).

**5. Pooled data analysis**

All three experiments showed that imagining the to-be-detected stimulus during external detection leads to a higher likelihood of reporting stimulus presence. In contrast, detection sensitivity, measured by d’, showed a significant decrease due to imagery only in Experiment 1. Furthermore, higher imagery vividness was associated with a more liberal detection criterion but was not related to d’. To further investigate the differences between (congruent) imagery and no-imagery on criterion and d’, we pooled together the data from these conditions from all three experiments for follow-up analyses.

As expected, the pooled analysis also revealed a significant decrease in criterion due to imagery (*t*(229) = -9.72, *p* = 6.81e-19), with a medium effect size (Cohen’s d = 0.532). Furthermore, when pooling all data together, there was also a significant decrease in detection sensitivity (d’) due to imagery *t*(229) = -3.67, *p* = 0.0003), with a small effect size (Cohen’s d = 0.230). If the decrease in criterion and the decrease in d’ were driven by the same underlying mechanisms, we might expect them to correlate between participants. However, there was no significant correlation between the decrease in criterion and the decrease in d’ (*r* = 0.059, *p* = 0.377), suggesting that these effects may have different causes. Furthermore, in this pooled analysis, imagery vividness significantly correlated with the shift in criterion (*r* = -0.215, *p* = 0.001) but not with the shift in d’ (*r* = -0.079, *p* = 0.231).

Taken together, imagery seems to exert differential influences on detection criterion and sensitivity. The effect on criterion is related to imagery vividness and might therefore be mostly due to imagery-specific processes. In contrast, the decrease in detection sensitivity might reflect a decrease in attentional resources dedicated to external detection due to the dual-task nature of the imagery compared to the no-imagery condition.

**Discussion**

In the current study we investigated how mental imagery affects detection of external stimuli in a situation where both are made to be similar in terms of content and signal strength. Participants imagined oriented gratings while at the same time externally detecting these gratings at threshold. In the first experiment, we showed that imagery led to more liberal detection criteria. In a second experiment, we replicated this finding and showed that this effect was independent of expectation. Finally, in a third experiment, we showed that the decrease in detection criterion was only observed when the imagined and external stimuli were congruent. Furthermore, in all three experiments we found a correlation between imagery vividness and perceptual detection, irrespective of the specific condition. When pooling the data of all experiments together, we also found a small decrease in d’. However, contrary to the criterion effects, this change in d’ was not related to imagery vividness and might therefore be due to distraction caused by the dual-task nature of imagery trials. Together, these results suggest that imagined stimuli can sometimes be confused for real ones, leading to an increase in presence reports when imagining the to-be-detected stimulus.

There are a number of possible mechanistic accounts of the observed effects of imagery on detection criterion. A decrease in criterion might be caused by an increase in low-level sensory signals or by a lowered decision threshold (Bang & Rahnev, 2017; Choe, Blake, & Lee, 2014; Kok, Mostert, & de Lange, 2017). A low-level sensory effect could further be caused by two mechanisms: increases in stimulus-specific signals could be caused either by an increase in baseline sensory activation or by an increase in sensitivity (contrast gain) for stimulus-like external signals (Reynolds & Heeger, 2009). Decreases in criterion induced by expectation have recently been explained as involving changes in stimulus-specific gain (Wyart, Nobre, & Summerfield, 2012; Yon, Zainzinger, de Lange, Eimer, & Press, 2020), suggesting that a similar mechanism may be in play here. According to these accounts, expectation makes stimulus-specific sensory units more sensitive to incoming signals, in turn making the detection threshold more likely to be breached. During stimulus-absent trials, this change in sensitivity selectively increases stimulus-specific signals in noise, allowing signal-like fluctuations in noise to sometimes cross the detection threshold, leading to an increase in false alarms. During stimulus-present trials, the same bias amplifies real signals, leading to an increase in hits. Importantly, this mechanism accounts for criterion shifts in terms of changes in the processing of external signals.

In contrast, an explanation in line with source confusion would predict that imagery internally generates stimulus-specific sensory activation in the absence of changes in sensory gain (i.e., a baseline shift), and that such internal generation is occasionally mistakenly inferred to be due to an external stimulus. During stimulus-absent trials, this internally generated sensory activation then sometimes crosses the detection threshold, leading to an increase in false alarms. During stimulus-present trials, signals that would have been too weak to cross the threshold in the absence of imagery, are now boosted by the addition of internally-generated activation, leading to an increase in hits. Neuroimaging studies showing that imagery activates sensory representations in a perception-like way are in favour of an explanation in terms of source confusion (Dijkstra et al., 2019; Pearson, 2019); however, the neurophysiological interpretation of neuroimaging measurements is not unambiguous (Goense & Logothetis, 2008). Furthermore, in contrast to top-down signals generated by expectations, internally generated signals during imagery are generally strong enough to lead to a visual experience in the absence of external input (Kosslyn, Ganis, & Thompson, 2001). Fully disentangling these two accounts of the effect of imagery on perceptual detection may be possible in future studies by using drift diffusion modelling (Yon et al., 2020) or by varying stimulus energy in a systemic way over trials (Wyart et al., 2012).

Finally, imagery causing a decrease in decision threshold would not be in line with source confusion. In the second experiment we also found that if participants were aware that imagery might influence detection, the imagery effect was bigger. This finding could mean that the belief that imagery influences detection made participants respond accordingly, suggesting our results might (partly) reflect demand characteristics (e.g. Lush, 2020). Demand characteristics might have even influenced the congruency effect in Experiment 3 if participants realized we expected incongruent imagery to influence detection differently from congruent imagery. However, it seems unlikely that demand characteristics could have explained the observed relationship with imagery vividness. Previous suggestions that imagery vividness might be related to demand characteristics (Hoen, 1976; Predebon & Wenderoth, 1985; Vesta et al., 1971) has led to a change in the framing of vividness questionnaires. In the current version of the VVIQ used at the start of Experiment 1 it is explicitly stated that it is "not necessarily desirable to experience imagery or, if you do, to have more vivid imagery.” (Marks, 1995). Furthermore, subjective imagery vividness ratings such as those collected here have been shown to correlate with objective measures of imagery strength as well as with neural processing in sensory areas (Cui, Jeter, Yang, Montague, & Eagleman, 2007; Dijkstra, Bosch, & van Gerven, 2017; Lee, Kravitz, & Baker, 2012; Pearson, Rademaker, & Tong, 2011). Finally, a large body of literature has shown that mental imagery activates perception-like sensory signals (Albers, Kok, Toni, Dijkerman, & De Lange, 2013; Dijkstra, Bosch, & van Gerven, 2017; Lee, Kravitz, & Baker, 2012; Naselaris, Olman, Stansbury, Ugurbil, & Gallant, 2015; Pearson, Clifford, & Tong, 2008; Reddy, Tsuchiya, & Serre, 2010; Senden, Emmerling, van Hoof, Frost, & Goebel, 2019). Therefore, while we cannot fully rule out demand characteristics as a contributing factor for our results we believe that our findings are more likely to reflect sensory effects. In this context, the relationship between awareness and imagery effect size can be explained by participants with stronger imagery effects being more likely to become aware of them.

There are substantial differences between individuals in the vividness of mental imagery (Galton, 1880; Pearson & Kosslyn, 2015). In the current study, we did not observe any relation between our experimental and questionnaire measures of imagery vividness or hallucination proneness. However, in all three experiments, we did observe a strong negative correlation between imagery vividness ratings during the practice trials and general detection criterion. In other words, more vivid imagery was associated with an increased tendency to report the presence of external stimuli. This finding suggests that deciding about internal stimulus presence (imagery vividness) and external stimulus presence (detection criterion) might rely on a common mechanism (Fazekas, Nemeth, & Overgaard, 2020). This is in line with studies showing a positive correlation between imagery vividness and hallucinations (Matthews, Collins, Thakkar, & Park, 2014; Sack, Van De Ven, Etschenberg, Schatz, & Linden, 2005; Salge, Pollmann, & Reeder, 2020; Shine et al., 2015; Stephan-Otto et al., 2017). Similar to our main finding, the relation between imagery vividness and detection criterion can be explained as a low-level sensory effect or a high-level decision effect. A sensory explanation would be that both imagery vividness and external detection threshold are related to visual cortex excitability. In line with this, a recent study has shown that resting state excitability of primary visual cortex is related to visual imagery strength (Rebecca Keogh, Bergmann, & Pearson, 2020). Alternatively, it could be that both vividness and criterion reflect a participant’s decision threshold for deciding whether they ‘see’ something, irrespective of whether that something is imagined or perceived. These two hypotheses could be teased apart in future research by investigating whether both measures corelate with low-level visual cortex activation or higher-level fronto-parietal activation.

Interestingly, in all three experiments we only observed one direction of source confusion: imagined stimuli being mistaken for real, leading to a decrease in detection criterion. Early studies of mental imagery showed that when participants were asked to imagine certain objects and describe what they experienced, external presentation of these same objects at threshold was often missed (Perky, 1910). This observation, called the Perky effect, was interpreted as perception being mistaken for imagery, corresponding to an imagery-induced increase in criterion (Perky, 1910; Segal & Nathan, 1964; Segal & Glicksman, 1967). One difference between those studies and the current experiments is that participants were unaware that external stimuli would be presented. However, one study did inform participants that real stimuli might be shown in some trials and still observed a slight increase in criterion (Segal & Fusella, 1969). Furthermore, in the current study, we still found an increase in presence reports when the expectation of external stimuli was low. Another difference is that the focus of the task in previous studies was on describing imagery accurately, with external detection treated as a less important side task. Indeed, studies in which the focus was on external detection (as here) generally reported a decrease in criterion (Segal & Fusella, 1970; Moseley, Smailes, Ellison, & Fernyhough, 2016; Saad & Silvanto, 2013). Together, this suggests that the direction of source confusion might be influenced by whether attention is directed inwards (imagery) or outwards (detection). Future research should investigate this by characterizing the effect of imagery on detection criterion during manipulations of the focus of attention.

Another important issue is whether the imagery effects observed here could be (partly) due to imagery increasing visual attention to the external stimulus. Indeed, spatial attention has also been shown to sometimes decrease criterion (Hawkins et al., 1999; Downing et al., 1988; Yon et al., 2020), leading to more presence responses. Furthermore, similar to imagery, attention increases sensory activation for the attended stimulus (Ling & Carrasco, 2006; Ling et al., 2009; Liu et al., 2007; Lui et al., 2005), even in the absence of external input (Chelazzi et al., 1998; Chelazzi et al., 1993; Tanaka, 1996; Peelen & Kastner, 2011). A recent paper directly investigated the relationship between mental imagery and (preparatory) attention by testing whether they resulted in similar effects on subsequent ambiguous perception (Keogh & Pearson, 2021). The results indicate that preparatory attention (i.e. the creation of an attentional template) in the absence of external input is similar to imagery, whereas feature-based attention in the presence of external input seems to rely on different mechanisms (Keogh & Pearson, 2021). This overlap between imagery and attentional templates might explain the large decrease in d’ during incongruent imagery in our study, because in this condition the attentional template relevant for the detection task and the mental image are competing (Olivers et al., 2011). However, we note that here, we also observed a slight decrease in d’ in the congruent condition. In contrast, congruent (preparatory) attention reliably increases d’ (e.g. Wyart et al., 2012; Stein & Peelen, 2015; for a review, see Carrasco, 2011), suggesting dissociable mechanisms. That is, attention is known to improve sensitivity to congruent sensory signals, whereas imagery here leads to a more liberal, but less sensitive response to congruent sensory signals. Furthermore, another important difference between (preparatory) attention and imagery is that, contrary to attention, imagery is associated with a conscious experience of the imagined stimulus (Keogh & Pearson, 2021) which could cross an external detection threshold, leading to internal signals being confused for perception. Taken together, these considerations suggest our results cannot be explained by imagery merely increasing visual attention. However, further research is necessary to fully characterize the relationship between imagery and attention.

Taken together, we have shown that imagining a stimulus while detecting that same stimulus at threshold leads to an increased tendency to say that external stimuli are present. This effect may be explained by internally generated sensory signals during imagery being erroneously attributed to an external source. This is in line with the idea that the neural correlates of imagined and perceived stimuli are highly similar such that perceptual reality monitoring is a non-trivial process that is prone to error in specific circumstances (Dijkstra et al., 2019). Our study encompasses three separate behavioral experiments, each of which builds on the previous one by ruling out alternative explanations of the results. For instance, we show that the robust decrease in perceptual detection criterion observed in Experiment 1 is not influenced by expectation (Experiment 2) and is stimulus-specific (Experiment 3). However, our study also raises several questions for further work, and ruling out alternative explanations of these results will require the development of novel behavioral paradigms, possibly combined with neuroimaging. Specifically, future studies should disentangle the contributions of imagery-induced sensory activation, sensory gain and decisional or response biases. The current results emphasize that our conscious perceptual experience arises from a complex interplay between external and internal signals and that sometimes the source of these sensory signals is unclear, leading to confusion between internally generated imagination and externally generated reality.

**Competing interests**

The authors declare that there are no competing interests.

**Acknowledgements**

N.D. is supported by a Rubicon grant from the Netherlands Organization for Scientific Research (NOW) [019.192SG.003], M.M. is supported by a Graduate Research Scholarship and an Overseas Research Scholarship from UCL, P.K. is supported by a Sir Henry Dale Fellowship jointly funded by the Wellcome Trust and the Royal Society [218535/Z/19/Z], S.M.F. is supported by a Wellcome/Royal Society Sir Henry Dale Fellowship [206648/Z/17/Z] and a Philip Leverhulme Prize from the Leverhulme Trust. The Wellcome Centre for Human Neuroimaging is supported by core funding from the Wellcome Trust [206648/Z/17/Z].

**Supplementary material**

All data, analysis and experimental code supporting this manuscript can be found at <https://github.com/NadineDijkstra/IMADET>.

**References**

Albers, A. M., Kok, P., Toni, I., Dijkerman, H. C., & De Lange, F. P. (2013). Shared representations for working memory and mental imagery in early visual cortex. *Current Biology*, *23*, 1427–1431. https://doi.org/10.1016/j.cub.2013.05.065

Bang, J. W., & Rahnev, D. (2017). Stimulus expectation alters decision criterion but not sensory signal in perceptual decision making. *Scientific Reports*, *7*(1), 1–12. https://doi.org/10.1038/s41598-017-16885-2

Bettencourt, K. C., & Xu, Y. (2015). Decoding the content of visual short-term memory under distraction in occipital and parietal areas. Nature Neuroscience, 19(1), 150–157. https://doi.org/10.1038/nn.4174

Carrasco, M. (2011). Visual attention: The past 25 years. *Vision Research*, 51, 1484–1525. https://doi.org/10.1016/j.visres.2011.04.012

Chang, A. Y. C., Kanai, R., & Seth, A. K. (2015). Cross-modal prediction changes the timing of conscious access during the motion-induced blindness. *Consciousness and Cognition*, *31*, 139–147. https://doi.org/10.1016/j.concog.2014.11.005

Chelazzi, L., Miller, E. K., Duncan, J., & Desimone, R. (1993). A neural basis for visual search in inferior temporal cortex. *Nature*, 363, 345-7. https://doi.org/10.1038/363345a0

Chelazzi, L., Duncan, J., Miller, E. K., & Desimone, R. (1998). Responses of neurons in inferior temporal cortex during memory-guided visual search. J*ournal of Neurophysiology*, 80(6), 2918-40. https://doi.org/10.1152/jn.1998.80.6.2918

Chiou, R., Rich, A. N., Rogers, S., & Pearson, J. (2018). Exploring the functional nature of synaesthetic colour: Dissociations from colour perception and imagery. *Cognition*, *177*, 107–121. https://doi.org/10.1016/J.COGNITION.2018.03.022

Choe, K. W., Blake, R., & Lee, S. H. (2014). Dissociation between neural signatures of stimulus and choice in population activity of human V1 during perceptual decision-making. *Journal of Neuroscience*, *34*(7), 2725–2743. https://doi.org/10.1523/JNEUROSCI.1606-13.2014

de Leeuw, J. R. (2015). jsPsych: A JavaScript library for creating behavioral experiments in a Web browser. *Behavior Research Methods*. https://doi.org/10.3758/s13428-014-0458-y

Delamillieure, P., Doucet, G., Mazoyer, B., Turbelin, M.-R., Delcroix, N., Mellet, E., … Joliot, M. (2010). The resting state questionnaire: An introspective questionnaire for evaluation of inner experience during the conscious resting state. *Brain Research Bulletin*, *81*(6), 565–573. https://doi.org/10.1016/J.BRAINRESBULL.2009.11.014

Denison, R. N., Piazza, E. A., & Silver, M. A. (2011). Predictive Context Influences Perceptual Selection during Binocular Rivalry. *Frontiers in Human Neuroscience*, *5*, 166. https://doi.org/10.3389/fnhum.2011.00166

Dhand, N. K., & Khatkar, M. S. (2014). Statulator: An online statistical calculator. Sample Size Calculator for Comparing Two Paired Proportions. . Accessed 21 November 2017 at Http://Statulator.Com/SampleSize/Ss2PP.Html.

Dijkstra, N., Bosch, S. E., & van Gerven, M. A. J. (2017). Vividness of visual imagery depends on the neural overlap with perception in visual areas. *Journal of Neuroscience*, *37*(5). https://doi.org/10.1523/JNEUROSCI.3022-16.2016

Dijkstra, N., Hinne, M., Bosch, S. E., & van Gerven, M. A. J. (2019). Between-subject variability in the influence of mental imagery on conscious perception. *Scientific Reports*, 9. https://doi.org/10.1038/s41598-019-52072-1

Dijkstra, Nadine, Bosch, S. E., & van Gerven, M. A. J. (2019). Shared neural mechanisms of visual perception and imagery. *Trends in Cognitive Sciences*, 23(5), 423-434. https://doi.org/10.1016/j.tics.2019.02.004

Downing, C. J. (1988). Expectancy and Visual-Spatial Attention: Effects on Perceptual Quality. Journal of Experimental Psychology: Human Perception and Performance, 14(2), 188–202. https://doi.org/10.1037/0096-1523.14.2.188

Fazekas, P., Nemeth, G., & Overgaard, M. (2020). Perceptual representations and the vividness of stimulus-triggered and stimulus-independent experiences. *Perspectives on Psychological Science*. https://doi.org/10.1177/1745691620924039

Galton, F. (1880). Statistics of mental imagery. *Mind*, *os*-*V*(19), 301–318. https://doi.org/10.1093/mind/os-V.19.301

Goense, J. B. M., & Logothetis, N. K. (2008). Neurophysiology of the BOLD fMRI Signal in Awake Monkeys. *Current Biology*, *18*(9), 631–640. https://doi.org/10.1016/j.cub.2008.03.054

Hautus, M. J. (1995). Corrections for extreme proportions and their biasing effects on estimated values of d′. *Behavior Research Methods, Instruments, & Computers*, *27*(1), 46–51. https://doi.org/10.3758/BF03203619

Hawkins, H. L., Hillyard, S. A., Luck, S. J., Mouloua, M., Downing, C. J., & Woodward, D. P. (1990). Visual Attention Modulates Signal Detectability. Journal of Experimental Psychology: Human Perception and Performance, 16(4), 802–811. https://doi.org/10.1037/0096-1523.16.4.802

Hohwy, J., Roepstorff, A., & Friston, K. (2008). Predictive coding explains binocular rivalry: An epistemological review. *Cognition*, *108*(3), 687–701. https://doi.org/10.1016/j.cognition.2008.05.010

Ingleby, J. D. (1967). Signal detection theory and psychophysics. *Journal of Sound and Vibration*. https://doi.org/10.1016/0022-460x(67)90197-6

Keogh, R, & Pearson, J. (2011). Mental imagery and visual working memory. *PLoS ONE*, *6*, e29221. https://doi.org/10.1371/journal.pone.0029221

Keogh, R., Bergmann, J., & Pearson, J. (2020). Cortical excitability controls the strength of mental imagery. *ELife*, *9*. https://doi.org/10.7554/eLife.50232

Keogh, R., & Pearson, J. (2014). The sensory strength of voluntary visual imagery predicts visual working memory capacity. *Journal of Vision*, *14*(12), 1427–1431. https://doi.org/10.1167/14.12.7

Keogh, R., & Pearson, J. (2017). The perceptual and phenomenal capacity of mental imagery. *Cognition*, *162*, 124–132. https://doi.org/10.1016/j.cognition.2017.02.004

Keogh, R., & Pearson, J. (2021). Attention driven phantom vision: measuring the sensory strength of attentional templates and their relation to visual mental imagery and aphantasia. Philosophical Transactions of the Royal Society B: Biological Sciences, 376(1817), 20190688. https://doi.org/10.1098/rstb.2019.0688

Kok, P., Mostert, P., & de Lange, F. P. (2017). Prior expectations induce prestimulus sensory templates. *Proceedings of the National Academy of Sciences*, 201705652. https://doi.org/10.1073/pnas.1705652114

Kosslyn, S. M., Ganis, G., & Thompson, W. L. (2001). Neural Foundations of Imagery. *Nature Reviews Neuroscience*, *2*(9), 635–642. https://doi.org/10.1038/35090055

Lange, K., Kühn, S., & Filevich, E. (2015). "Just Another Tool for Online Studies” (JATOS): An Easy Solution for Setup and Management of Web Servers Supporting Online Studies. *PLOS ONE*, *10*(6), e0130834. https://doi.org/10.1371/journal.pone.0130834

Launay, G., & Slade, P. (1981). The measurement of hallucinatory predisposition in male and female prisoners. *Personality and Individual Differences*, *2*(3), 221–234. https://doi.org/10.1016/0191-8869(81)90027-1

Lee, S.-H., Kravitz, D. J., & Baker, C. I. (2012). Disentangling visual imagery and perception of real-world objects. *NeuroImage*, *59*(4), 4064–4073. https://doi.org/10.1016/j.neuroimage.2011.10.055

Ling, S., & Carrasco, M. (2006). Sustained and transient covert attention enhance the signal via different contrast response functions. Vision Research, 46(8–9), 1210–1220. https://doi.org/10.1016/j.visres.2005.05.008

Ling, S., Liu, T., & Carrasco, M. (2009). How spatial and feature-based attention affect the gain and tuning of population responses. Vision Research, 49(10), 1194–1204. https://doi.org/10.1016/j.visres.2008.05.025

Liu, T., Larsson, J., & Carrasco, M. (2007). Feature-Based Attention Modulates Orientation-Selective Responses in Human Visual Cortex. Neuron, 55(2), 313–323. https://doi.org/10.1016/j.neuron.2007.06.030

Liu, T., Pestilli, F., & Carrasco, M. (2005). Transient attention enhances perceptual performance and fMRI response in human visual cortex. Neuron, 45(3), 469–477. https://doi.org/10.1016/j.neuron.2004.12.039

Marks, D F. (1973). Visual imagery differences in the recall of pictures. *British Journal of Psychology (London, England : 1953)*, *64*, 17–24. https://doi.org/10.1111/j.2044-8295.1973.tb01322.x

Marks, David F. (1995). New directions for mental imagery research. *Journal of Mental Imagery*, *19*(3–4), 153–167. https://doi.org/10.1177/0963721414532287

Matthews, N. L., Collins, K. P., Thakkar, K. N., & Park, S. (2014). Visuospatial imagery and working memory in schizophrenia. *Cognitive Neuropsychiatry*, *19*(1), 17–35. https://doi.org/10.1080/13546805.2013.779577

McCarthy-Jones, S., & Fernyhough, C. (2011). The varieties of inner speech: Links between quality of inner speech and psychopathological variables in a sample of young adults. *Consciousness and Cognition*, *20*(4), 1586–1593. https://doi.org/10.1016/j.concog.2011.08.005

Morrison, A. P., Wells, A., & Nothard, S. (2000). Cognitive factors in predisposition to auditory and visual hallucinations. *British Journal of Clinical Psychology*, *39*(1), 67–78. https://doi.org/10.1348/014466500163112

Moseley, P., Smailes, D., Ellison, A., & Fernyhough, C. (2016). The effect of auditory verbal imagery on signal detection in hallucination-prone individuals. *Cognition*, *146*, 206–216. https://doi.org/10.1016/j.cognition.2015.09.015

Naselaris, T., Olman, C. A., Stansbury, D. E., Ugurbil, K., & Gallant, J. L. (2015). A voxel-wise encoding model for early visual areas decodes mental images of remembered scenes. *NeuroImage*, *105*, 215–228. https://doi.org/10.1016/j.neuroimage.2014.10.018

Okada, H., & Matsuoka, K. (1992). Effects of auditory imagery on the detection of a pure tone in white noise: experimental evidence of the auditory Perky effect. *Perceptual and Motor Skills*, *74*(2), 443–448. https://doi.org/10.2466/pms.1992.74.2.443

Olivers, C. N. L., Peters, J., Houtkamp, R., & Roelfsema, P. R. (2011). Different states in visual working memory: When it guides attention and when it does not. Trends in Cognitive Sciences, 5, 327–334. https://doi.org/10.1016/j.tics.2011.05.004

Pearson, J. (2019). The human imagination: the cognitive neuroscience of visual mental imagery. *Nature Reviews Neuroscience, 20,* 624-634. https://doi.org/10.1038/s41583-019-0202-9

Pearson, J., Clifford, C. W. G., & Tong, F. (2008). The functional impact of mental imagery on conscious perception. *Current Biology*, *18*(13), 982–986. https://doi.org/10.1016/j.cub.2008.05.048

Pearson, J., & Kosslyn, S. M. (2015). The heterogeneity of mental representation: Ending the imagery debate. *Proceedings of the National Academy of Sciences of the United States of America*, *112*(33), 10089–10092. https://doi.org/10.1073/pnas.1504933112

Peelen, M. V., & Kastner, S. (2011). A neural basis for real-world visual search in human occipitotemporal cortex, 108, 12125-12130. *Proceedings of the National Academy of Sciences of the United States of America*. https://doi.org/10.1073/pnas.1101042108

Perky, C. W. (1910). An Experimental Study of Imagination. *The American Journal of Psychology*, *21*(3), 422. https://doi.org/10.2307/1413350

Pinto, Y., van Gaal, S., de Lange, F. P., Lamme, V. A. F., & Seth, A. K. (2015). Expectations accelerate entry of visual stimuli into awareness. *Journal of Vision*, *15*(8), 13–13. https://doi.org/10.1167/15.8.13

Rademaker, R. L., Chunharas, C., & Serences, J. T. (2019). Coexisting representations of sensory and mnemonic information in human visual cortex. Nature Neuroscience, 22(8), 1336–1344. https://doi.org/10.1038/s41593-019-0428-x

Reddy, L., Tsuchiya, N., & Serre, T. (2010). Reading the mind’s eye: decoding category information during mental imagery. *NeuroImage*, *50*(2), 818–825. https://doi.org/10.1016/j.neuroimage.2009.11.084

Reynolds, J. H., & Heeger, D. J. (2009, January 29). The Normalization Model of Attention. Neuron, 61, 168–185. https://doi.org/10.1016/j.neuron.2009.01.002

Saad, E., & Silvanto, J. (2013). Contrast and strength of visual memory and imagery differentially affect visual perception. *PLoS ONE*, *8*(12). https://doi.org/10.1371/journal.pone.0084827

Sack, A. T., Van De Ven, V. G., Etschenberg, S., Schatz, D., & Linden, D. E. J. (2005). Enhanced vividness of mental imagery as a trait marker of schizophrenia? *Schizophrenia Bulletin*. https://doi.org/10.1093/schbul/sbi011

Salge, J. H., Pollmann, S., & Reeder, R. R. (2020). Anomalous visual experience is linked to perceptual uncertainty and visual imagery vividness. *Psychological Research*, (0123456789). https://doi.org/10.1007/s00426-020-01364-7

Segal, S. J., & Fusella, V. (1969). Effects of imaging of signal-to-noise ratio, with varying signal conditions. *British Journal of Psychology (London, England : 1953)*, *60*(4), 459–464. https://doi.org/10.1111/j.2044-8295.1969.tb01219.x

Segal, S. J., & Fusella, V. (1970). Influence of imaged pictures and sounds on detection of visual and auditory signals. *Journal of Experimental Psychology*, *83*(3 PART 1), 458–464. https://doi.org/10.1037/h0028840

Segal, S. J., & Nathan, S. (1964). The Perky Effect: Incorporation of an External Stimulus into an Imagery Experience under Placebo and Control Conditions. *Perceptual and Motor Skills*, *18*(2), 385–395. https://doi.org/10.2466/pms.1964.18.2.385

Segal, S. J., & Glicksman, M. (1967). Relaxation and the Perky effect: the influence of body position on judgments of imagery. *The American Journal of Psychology*, *80*(2), 257–262. https://doi.org/10.2307/1420985

Senden, M., Emmerling, T. C., van Hoof, R., Frost, M. A., & Goebel, R. (2019). Reconstructing imagined letters from early visual cortex reveals tight topographic correspondence between visual mental imagery and perception. *Brain Structure and Function*, *224*(3), 1167–1183. https://doi.org/10.1007/s00429-019-01828-6

Sherwood, R., & Pearson, J. (2010). Closing the mind’s eye: incoming luminance signals disrupt visual imagery. *PloS One*, *5*(12), e15217. https://doi.org/10.1371/journal.pone.0015217

Shine, J. M., Keogh, R., O’Callaghan, C., Muller, A. J., Lewis, S. J. G., & Pearson, J. (2015). Imagine that: Elevated sensory strength of mental imagery in individuals with Parkinson’s disease and visual hallucinations. *Proceedings of the Royal Society B: Biological Sciences*. https://doi.org/10.1098/rspb.2014.2047

Stein, T., & Peelen, M. V. (2015). Content-specific expectations enhance stimulus detectability by increasing perceptual sensitivity. *Journal of Experimental Psychology: General*, *144*(6), 1089–1104. https://doi.org/10.1037/xge0000109

Stephan-Otto, C., Siddi, S., Senior, C., Cuevas-Esteban, J., Cambra-Martí, M. R., Ochoa, S., & Brébion, G. (2017). Remembering verbally-presented items as pictures: Brain activity underlying visual mental images in schizophrenia patients with visual hallucinations. *Cortex*, *94*, 113–122. https://doi.org/10.1016/j.cortex.2017.06.009

Tanaka, K. (1996). Inferotemporal cortex and object vision. Annual Review of Neuroscience. https://doi.org/10.1146/annurev.ne.19.030196.000545

Witt, J. K., Taylor, J. E. T., Sugovic, M., & Wixted, J. T. (2015). Signal detection measures cannot distinguish perceptual biases from response biases. *Perception*, *44*(3), 289–300. https://doi.org/10.1068/p7908

Wyart, V., Nobre, A. C., & Summerfield, C. (2012). Dissociable prior influences of signal probability and relevance on visual contrast sensitivity. *Proceedings of the National Academy of Sciences of the United States of America*, *109*(9), 3593–3598. https://doi.org/10.1073/pnas.1120118109

Yon, D., Zainzinger, V., de Lange, F., Eimer, M., & Press, C. (2020). Action biases perceptual decisions towards expected outcomes. *Journal of Experimental Psychology: General*. https://doi.org/10.31234/osf.io/3zp8n
